# Mindfulness Training in Military Settings: Emerging Evidence and Best-Practice Guidance

**DOI:** 10.1007/s11920-025-01608-6

**Published:** 2025-06-03

**Authors:** Amishi P. Jha, Mary K. Izaguirre, Amy B. Adler

**Affiliations:** 1 Department of Psychology, University of Miami, Coral Gables, FL 33146 USA; 2 Medical Command, US Army, Falls Church, VA 22042 USA; 3 Center for Military Psychiatry and Neuroscience, Walter Reed Army Institute of Research, Silver Spring, MD 20910 USA; 4 5665 Ponce de Leon, Coral Gables, FL 33146 USA

**Keywords:** Mindfulness training, Implementation, Attention, Performance

## Abstract

**Purpose of Review:**

Evidence suggests that mindfulness training (MT) may protect and strengthen military service members’ attentional control functions, improving their performance and holistic fitness as they face the modern-day battlefield. Yet, implementation challenges must be addressed to realize MT’s benefits consistently and at scale.

**Recent Findings:**

Despite heterogeneity in MT program content, evaluation metrics, and participants’ military career stages across studies, recent findings suggest that MT may bolster performance, cognitive functions, psychological well-being, and social relationships. Additionally, implementation factors such as daily practice, trainers’ familiarity with the military, and course content influence the extent of benefits.

**Summary:**

Attentional control is critical for effective performance, yet vulnerable to compromise in high-demand cohorts, such as military service members. MT not only targets and strengthens military service members’ attentional control but also enhances other aspects of their functioning. Despite recognized hurdles, best practice guidance is emerging and continued research and efforts to implement MT in military settings are warranted.

## Introduction

*At the end of an early morning run, a platoon of service members gathers to hydrate and complete cool-down exercises. Then they all shift their attention to their unit leader, who plays a recording of a mindfulness exercise. Today, the recording guides them to anchor their attention on the physical sensations of the breath. They are reminded to focus on the breath, notice distractions that may pull focus away, and refocus. After 15 min, the recording ends, and they move on to tackle the demands of the duty day.*

This scene of soldiers engaging in a mindfulness exercise during their daily physical training (PT) might seem out of place with notions of military fitness. Strenuous aerobic exercise — now considered a commonplace activity for physical fitness routines — was only introduced in the US military in the early 1970s, after the cardiovascular benefits of exercise were reported in large-scale military research studies [1, 2]. Soon after, PT was deemed essential for “total military preparedness” [2, pg. 139], leading to its system-wide adoption. Service members of all ranks received dedicated time for PT during the duty day, often being led through prescribed exercises, a practice that continues to this day. Fifty years later, mindfulness practice is at a similar crossroads, with military leaders weighing evidence to inform decisions on adoption at scale.

The emphasis on physical fitness to promote military preparedness is important, but offers an incomplete approach to readiness. Advancements in robotics, technology, and artificial intelligence are shifting the ratio of physical-to-cognitive task requirements during military operations, with physical demands set to be outpaced by cognitive demands by 2040 [3]. In addition, modern frameworks of comprehensive fitness in both military and civilian settings favor a holistic approach [4], emphasizing the importance of not only being physically fit, but also being cognitively sharp, socially connected, and emotionally balanced [5, 6].

One way to address this comprehensive approach to fitness is to consider the robust research literature examining the impact of mindfulness training (MT) in civilian cohorts, which reports MT-related benefits in performance, cognitive functioning, social connectedness, and psychological health [see 7 for review]. These multi-faceted benefits have contributed to MT’s growing popularity to support “holistic fitness” [8] and interest in its system-wide adoption in educational [9], healthcare [10], and workplace settings [11].

Compared to efforts in civilian settings worldwide [12], the research and application of MT in the military context is relatively recent [13, 14]. MT has been offered across a range of military settings, from entry-level training academies [15] and basic combat training [16, 17], to professional military education [18] and operational units [19–22]. While a variety of benefits have been reported [13], there is little consensus on best practices for system-wide MT implementation in the military.

In this article, we discuss emerging MT research in military cohorts to guide evidence-informed decisions regarding its military adoption. Specifically, we review: (1) MT’s promotion of holistic fitness, via strengthening of attentional control; (2) military MT studies and outcome domains; and (3) considerations for MT’s military implementation at scale.

## Mindfulness Training and Attentional Control

Mindfulness is a mental mode characterized by purposefully paying attention to present-moment experience without elaboration, editorializing, or emotional reactivity [23]. MT programs aim to cultivate this mental mode through repeated engagement in specific mindfulness exercises and in-class group discussions. MT has been viewed as a form of neurocognitive training with parallels to PT [24, 25]. During PT, body systems are targeted by engaging in specific physical exercises. To bolster physical *fitness* of a particular system (e.g., cardiovascular), physical exercises must be repeated consistently over time. Similarly, mindfulness exercises may target specific mental processes (e.g., attentional control) to bolster neurocognitive fitness. Practicing mindfulness exercises multiple times per week over a multi-week MT program interval may repeatedly engage these mental processes, strengthening selective focus, meta-awareness, and decentering over time [24, 26; see details below]. Moreover, there is growing evidence that MT improves neurocognitive functioning, as demonstrated by improvements in cognitive processes [27] and changes in the resting state functional connectivity of specific large-scale brain networks over MT training intervals [28, 29].

Reports from mindfulness practitioners offer valuable insights into the cognitive changes in information processing that occur with repeated mindfulness practice. One noteworthy example of MT’s capacity to bolster cognitive tendencies is from now-retired U.S. Army Lieutenant General (LTG) Walter Piatt who said, “Mindfulness exercises are like *push-ups for the mind*. They have helped me focus and stay focused on a situation or person more easily, without getting lost-in-thought or distracted by information being thrown at me. I can watch my mind and pull it back if it gets stuck in a memory or worry. I can drop the story of what I think should be happening, so I don’t become blind to what is actually happening.” [14, pg. 4].

To better understand how MT leads to these subjective changes, it’s essential to examine the mental processes that practitioners are guided to engage during mindfulness exercises. MT programs typically include two categories of formal exercises: focused attention (FA) and open monitoring (OM). In FA exercises, practitioners select a specific object of focus, such as breath-related sensations (e.g., the sensations of the breath at the nostrils or abdomen), and aim to maintain selective focus on this object, moment-by-moment. When they notice they are off-track, they are instructed to refocus back to the target object. FA is the exercise depicted at the start of this article. During OM, practitioners are instructed to maintain a receptive and open state, non-reactively monitoring the contents of conscious experience moment by moment, without sustaining focus on any specific object.

Recent theoretical models of MT [26] propose that successful engagement in mindfulness exercises requires execution of three core mental actions: (1) selective focus, processing specific information (such as an object or specific aspect of experience) while ignoring distractions [26]; (2) meta-awareness, monitoring and being aware of ongoing mental processes—such as sensing, feeling, and thinking—that are subjectively experienced [30, 31]; and (3) decentering, observing items that arise in the mind (feelings, thoughts, and memories) from a healthy psychological distance, recognizing them as mental events rather than direct reflections of reality [32].

These core mental actions, while essential for mindfulness exercises, are also useful during a variety of everyday situations. For example, when attempting to read a book in a noisy cafe, *selective focus* enables you to concentrate on the text while tuning out the clatter of dishes and background conversations. *Meta-awareness* comes into play when you suddenly realize your mind has drifted to thoughts about the weekend instead of following the words on the page. This recognition allows you to decide whether or not to refocus on reading. *Decentering*, in turn, helps create psychological distance from distracting thoughts or emotions. If an anxious thought arises—perhaps about an upcoming deadline—you can note, “I’m having an anxious thought” rather than becoming immersed in the emotion.

In addition to being relevant for ad hoc use during everyday circumstances, when these mental actions are stable, trait-like cognitive tendencies, they may be especially beneficial in high-stakes situations like those faced by military leaders. Indeed, this likely benefit aligns with the subjective experience described by LTG Piatt, in his observation of how MT helped him (see above). Specifically, selective focus was described as the ability to “focus and stay focused,” meta-awareness as the ability to “watch the mind,” and decentering as the ability to “drop the story.” Beyond this subjective account, numerous studies have reported improvements in validated measures of selective focus, meta-awareness, and decentering with MT [see 26, 27, 33]. Yet, much less is known about the mechanisms by which these improvements may occur.

**Fig. 1:**
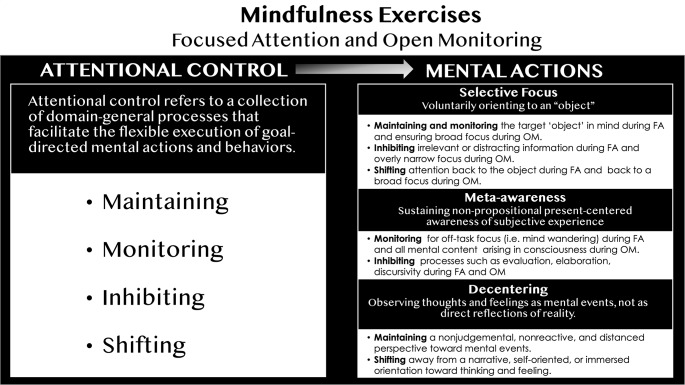
Mindfulness Exercises and Neurocognitive Training of Attentional Control. *Mindfulness exercises strengthen domain-general attentional control functions.* Practicing focused attention (FA) and open monitoring (OM) mindfulness exercises targets attentional control functions during the execution of three mental actions–selective focus, meta-awareness, and decentering. Repeatedly engaging in these exercises over multiple sessions offers a complex, variable, and challenging neurocognitive training environment which strengthens attentional control over time

In Fig. 1, we depict a mechanistic framework, proposing that MT not only strengthens core mental actions (selective focus, meta-awareness, and decentering), but bolsters attentional control itself through its varied and repeated engagement during mindfulness exercises. Attentional control is the ability to regulate information processing in a flexible, goal-directed manner [34]. Because the objective of each mindfulness exercise—FA and OM—differs, the way in which core mental actions (selective focus, meta-awareness, and decentering) are engaged during each practice will also vary.

During FA, the goal is to engage selective focus so that information processing is constrained to a single object or aspect of experience (e.g., breath-related sensations) and not toward external distractions or internal distractions such as off-task thoughts or memories that may arise. Meta-awareness supports this goal by identifying when selective focus has drifted off-track, while decentering helps loosen the attentional grip of “sticky” mental content, such as a recurring worry. In contrast, during OM, selective focus remains broad and steady, encompassing all arising mental content. Meta-awareness continuously monitors this content without engaging in evaluation and elaboration, often referred to in the mindfulness literature as discursive thought. Decentering is proactively applied during OM, allowing thoughts and emotions to be observed nonjudgmentally and from a distance.

As depicted on the left side of Fig. 1, attentional control involves subordinate functions such as maintaining, monitoring, inhibiting, and shifting [35], which in turn support selective focus, meta-awareness, and decentering. Maintaining means sustaining focus on goal-relevant information over time. Monitoring allows for the continuous tracking of information processing, detecting distractions or lapses that may interfere with performance. Inhibiting suppresses the processing of irrelevant stimuli or impulses, preventing interference from competing information. Finally, shifting enables the flexible redirection of information processing back to a goal-aligned task set based on situational demands.

As described in the right column of Fig. 1, engagement of these subordinate functions during FA and OM ensures that information processing remains aligned with each practice’s goal. For example, selective focus requires *maintenance and monitoring* processes to be constrained to a single target during FA but broadly distributed across all arising mental phenomena during OM. Selective focus also requires *inhibiting* irrelevant information—such as any stimuli unrelated to the target during FA—and inhibiting an excessive narrowing of attention during OM. *Shifting* is required when selective focus has been erroneously directed toward irrelevant information in FA or when it has become overly narrow in OM. For meta-awareness, *monitoring* allows for the detection of off-task thoughts during FA and the continuous tracking of mental events that arise during OM. Additionally, meta-awareness requires *inhibiting* engagement in elaboration and evaluation during both FA and OM. And for decentering, attentional control is required to maintain a nonjudgmental and distanced perspective and to inhibit a self-oriented, immersive perspective during both FA and OM. Thus, our framework proposes that a variety of attentional control functions are engaged to support selective focus, meta-awareness, and decentering during mindfulness exercises.

Importantly, during MT programs, FA and OM are practiced repeatedly. The intention behind this requirement is not merely to improve one’s ability to practice, so that it becomes easier over repeated sessions. Instead, this time investment is made to benefit participants in their everyday lives. The underlying assumption is that MT will have *far transfer* effects—meaning that the benefits of repeatedly practicing mindfulness exercises will not be limited to benefitting subsequent practice sessions but will transfer to new tasks [36].

Research on cognitive training has identified key factors that promote far transfer of learning to new environments and activities. Specifically, training is most effective when the training environment is complex, variable, and challenging, and when the cognitive process being trained is engaged voluntarily, flexibly, and repeatedly [24]. In line with the mechanistic framework described in Fig. 1, we argue that attentional control is not only necessary for successfully engaging in FA and OM practices, but repeating mindfulness practices over a multi-week MT program fosters the training conditions that support far transfer.

Across practice sessions, participants experience a complex, variable, and ever-changing milieu of sensory input and mental activity while potentially facing challenges in following practice instructions. They must repeatedly engage multiple subordinate functions of attentional control—maintaining, monitoring, inhibiting, and shifting—in a goal-aligned manner, not only for FA or OM practice as a whole but also tailored to the specific mental action (e.g., selective focus) they are performing. Additionally, participants must determine when attentional control is necessary and engage it voluntarily, flexibly, and repeatedly—whether they are off-track during an FA practice or overly focused during an OM practice. Furthermore, they must initiate this control endogenously, relying on their own willful engagement without external prompting.

If MT does indeed provide a robust neurocognitive training environment for attentional control, we would expect to see MT-related improvements not only on laboratory-based attention tasks that share some features with mindfulness training exercises [see 37], but also in real-world tasks requiring attentional control [see 24]. These real-world tasks may share very few features with the training exercises.

MT-related improvements have been reported in a variety of laboratory-based tasks of attentional control. A recent meta-analysis of 111 studies, primarily involving civilian cohorts [27], reported significant small-to-moderate effect sizes on various laboratory-based tasks of attentional control, such as sustained attention and working memory accuracy. Additionally, a RAND report which reviewed a subset of MT research in civilians and military personnel concluded that overall effects of MT were small; however, the strength of evidence for attention, based on 23 studies, was regarded as moderate, suggesting that one of the key benefits of MT may be enhanced attentional control [13].

Beyond laboratory-based tasks, MT improvements have been demonstrated in various real-world tasks in civilians including workplace safety behaviors [38], driving skills [39], athletic performance [40], and academic achievement [41], all of which are known to depend on attentional control for success [34, 42]. MT-related enhancements have also been observed in military contexts, with increased marksmanship scores among service members [21]. These findings highlight how strikingly dissimilar the training and far transfer contexts can be. For a military service member, for example, while mindfulness exercises may be practiced while sitting quietly with eyes closed in a safe place, the performance benefits may occur in the chaotic and potentially dangerous context of training or combat.

In addition to these performance-related findings, there is evidence of MT-related functional brain changes within key nodes and networks of attentional control. For instance, relative to novices, mindfulness practitioners exhibit greater resting-state functional connectivity within the fronto-parietal control network—a large-scale network supporting attentional control [e.g., 43]. Furthermore, when novices undergo MT, this network shows enhanced modulation of other large-scale networks involved in salience detection and internally generated thought [see 28, 29, 33].

Together these findings support the view that MT provides a unique neurocognitive training environment that strengthens attentional control and benefits performance and brain fitness.

### The Relevance of MT in the Military

**Fig. 2:**
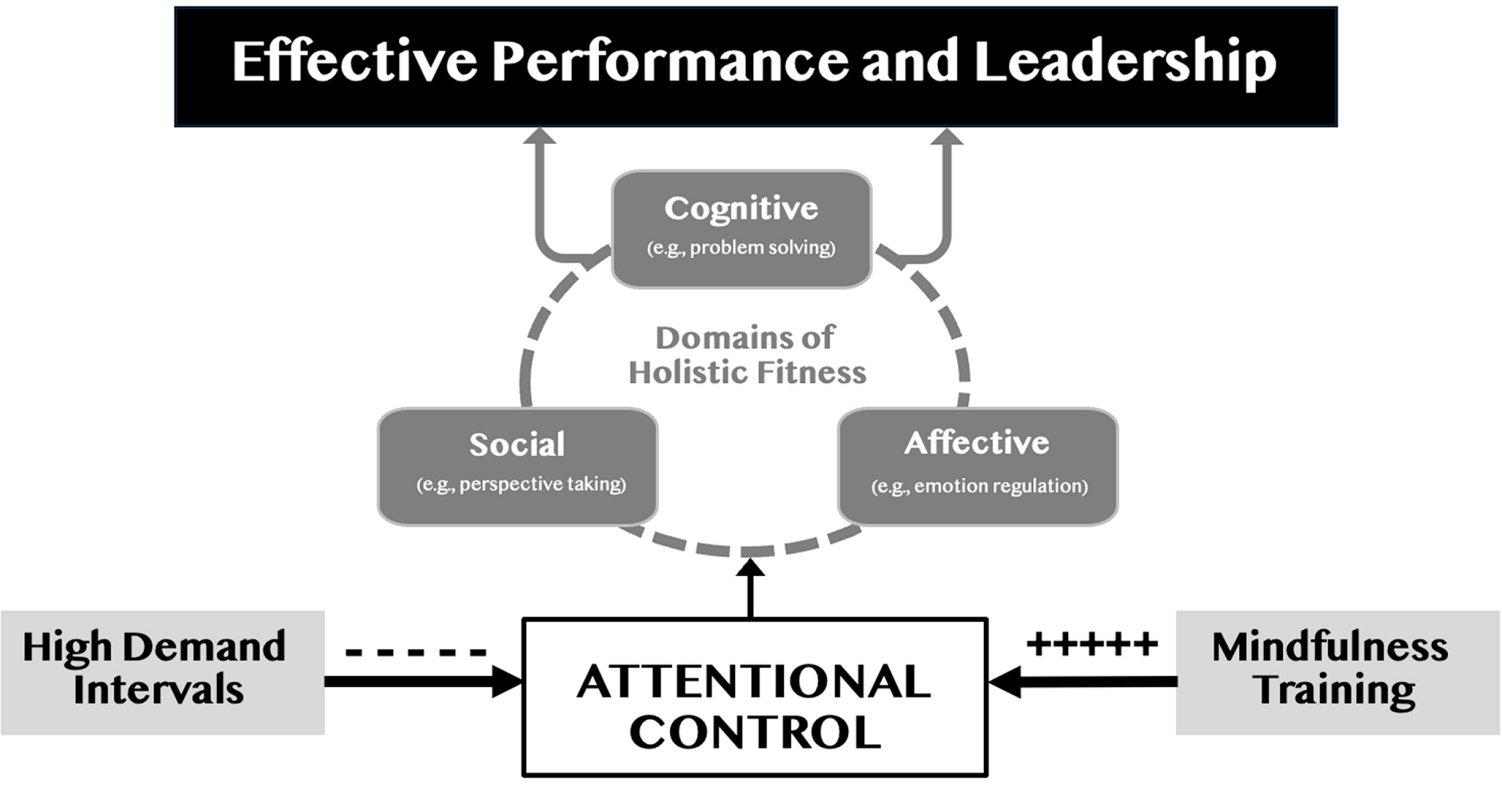
Attentional control is weakened by protracted intervals of high demand and strengthened by mindfulness training. Attentional control is conceptualized as a domain-general ‘fuel’ that supports a broad range of domain-specific processes such as cognitive, social, and emotional functions, which are critical for holistic fitness. The availability of attentional control resources determines the quality of holistic fitness, which, in turn, determines the success of the multi-domain processes they support, such as operational performance and leadership effectiveness

While mindfulness training strengthens attentional control, the high-demand conditions often endured in military service may make attentional control vulnerable to degradation—making service members among those who stand to benefit most from this training. In both training and deployed settings, risk of harm is amplified when service members are exposed to stress, threat, and lack of sleep, conditions which may compromise attentional control functions [44]. Moreover, this exposure may last for protracted time intervals. The deleterious consequences of high-demand intervals on attention-related outcomes have been reported across military contexts, including survival school [45] and pre-deployment training [46]. In addition, a greater number of performance errors have been reported in Army warfighters spending extended periods conducting simulated combat maneuvers [47] and other high-intensity military training [48]. Overall, such degradation potentially compromises individual service member fitness, as well as mission and leadership success (See Fig. 2).

Military culture is deeply hierarchical, and leaders play a critical role in strategic and tactical decisions. Leaders provide direction, support unit morale, and take care of subordinates [49]. Thus, when the attention of a service member in a leadership role is degraded, their decision making may falter, negatively impacting not only the individual, but also the unit and the mission. While leaders are important in a variety of organizations, in high-stakes occupations like the military, leaders are critical to survival.

Attentional control is essential for effective cognitive, social, and emotional functioning, particularly in the demanding environment of military service [14]. However, the intense pressures of real-world military operations can compromise attentional control [50]. The cognitive neuroscience literature has reported that diminished attentional control functions correspond with impaired decision-making [51, 52], diminished social abilities [53], and poor emotion regulation [54]. Various training approaches, such as computer-based cognitive task training [see 55], have been employed to strengthen attentional control among military personnel. Despite these efforts, most approaches to date have only achieved ‘near transfer,’ with benefits limited to the specific training context.

While MT holds promise for protecting and strengthening attentional control and producing far transfer of benefits, its effectiveness depends on the participant’s ability to practice in a way that aligns with the intended goals of mindfulness exercises. Unlike computer-based training, where the process is straightforward and externally driven, MT requires a nuanced understanding of how to practice correctly [56]. For example, during FA exercises, participants are instructed to observe the sensory experience of the breath without altering the breath’s natural rhythm. These instructions may appear to be similar to breathing exercises like deep breathing or box breathing. In box (or square) breathing, an individual typically breathes in for four seconds, holds for four seconds, exhales for four seconds, and holds again for four seconds. But in MT, the focus on the breath serves as an anchor for observing arising phenomena from a nonjudgmental perspective. The goal is to see clearly what is happening in the present moment, free from bias, interpretations, and distortions that can cloud judgment and compromise effective decision making. In contrast, practices like box breathing aim to change the breath to induce a calmer physiological state. While both methods are beneficial, their mechanisms and outcomes differ, making it crucial for participants to understand these distinctions to avoid confusion.

The effectiveness of MT significantly improves when led by a skilled trainer, who can correct misunderstandings and motivate participants to stay consistent, compared to a self-guided approach [27]. Trainers who understand the specific context in which participants operate, such as military environments, can further enhance the relevance and applicability of MT practices to participants’ professional and personal lives [50].

For MT to be effectively delivered in military settings, trainers must not only have direct experience with mindfulness practices but also be adept at linking practice-related concepts to the demands of military life [50, 57]. In contrast to extensive research on trainer competencies and MT delivery fidelity in civilian settings, there has been limited research on train-the-trainer programs specifically for military MT [see 16, 50]. Although further research is necessary, several MT studies have already been conducted with military cohorts, as reviewed in the next section.

## Mindfulness Training Research in the Military

Global interest in military MT is growing substantially. Studies have been conducted across a variety of military settings across several countries, including China [58], Germany [59–61], the Netherlands [22], New Zealand [18], Norway [62], the Republic of Korea [63], Taiwan [15], the United Kingdom [64], and the United States [e.g., 17, 65].

Research finds that MT benefits operationally relevant performance such as marksmanship [21] and reduces the risk of complex real-world failures [66]. It is also relevant to promoting holistic fitness. In terms of the cognitive domain, MT enhances and protects attention and working memory during high-demand intervals [see 67 for review]. For example, MT protected against declines in cognitive task performance in conventional forces during the interval prior to a combat deployment [see 68, 69]. More recently, elite forces who received MT demonstrated significant improvements in attention and working memory over a predeployment interval whereas those who received training-as-usual did not [see 65]. In contrast, one study [22] found that offering 5 to 10 min of MT over a 10-day interval did not result in changes in objective cognitive performance. Nevertheless, a recent internal meta-analysis of 6 studies with military cohorts found beneficial effects for working memory [70]. While effect sizes were small-to-moderate, they were comparable to those reported in civilian samples [27].

In terms of the social domain, studies with civilians demonstrate MT’s positive impact on marital relationships [71], team cohesion, team collaboration [72, 73], and quality of interpersonal communication [74]. There is also emerging evidence that MT leads to better cohesion in military service members (Gutierrez et al., under review) and British cadets [64].

While numerous studies have demonstrated clinical improvements associated with MT [75], MT also results in more positive emotion and well-being, and reduced negative emotion, perceived stress, and anxiety in psychologically healthy individuals [see 7 for review], including military cohorts [see 61, 62, 63]. Over high-demand intervals, MT also prevents a decline in mood and well-being [23, 76]. In addition, there is a dose-dependent relationship [23], with greater mood effects associated with more daily mindfulness exercise.

In terms of the physical domain, one study found soldiers assigned to receive a combination of mindfulness and yoga had fewer medical encounters for injury and less impact of pain on training than those assigned to training-as-usual [17]. While the relative benefits of MT and yoga could not be disentangled, both MT and yoga have been proposed to benefit attentional control [77]. Another study found that a mindfulness-based intervention led to fewer physical complaints and increased heart-rate variability in military personnel [59]. Furthermore, benefits of MT alone have been reported for physical pain [78] and biomarkers of stress [79]. Across each of these domains, there is evidence supporting MT’s benefits, highlighting its promise as an effective tool for holistic fitness in the military context (See Fig. 2).

Ensuring continued growth in understanding the multi-faceted benefits of MT for military cohorts will require that more studies engage high research standards no matter the domain being investigated. These standards include using well-established metrics with strong internal consistency, rigorous experimental designs such as randomized controlled trials (RCTs) with follow-up assessments, and the integration of both subjective and objective measures. Encouragingly, some studies have already begun to implement these high standards, offering valuable insights into MT’s impact. One such example is an 8-week MBSR study by Yan and colleagues [80], which examined psychological resilience in military medical students. The MBSR group’s resilience score, as measured by the Conner-Davidson Resilience Scale, was 17.1% higher than the control group at post-intervention and remained 23.3% above the control group at follow-up.

Beyond validated measures and robust RCT designs, it is also essential that studies incorporate active comparison groups rather than relying solely on no-training controls and integrate objective metrics alongside self-reported outcomes. Research has begun to embrace these refinements. For instance, a study in active-duty cohorts during a high-demand pre-deployment interval used a group-randomized design to compare MT with positivity training, ensuring both programs were matched for trainer expertise and duration [19]. Participants completed the well-validated Sustained Attention to Response Task (SART) to assess attentional performance. While both groups showed declines in task accuracy over this high demand interval, MT reduced the decline by approximately 80% compared to the positivity group, suggesting a protective effect of MT on sustained attention. In another well-designed study [20], both subjective and objective health measures were incorporated. In addition to self-reported outcomes, heart rate variability (HRV) and heart rate (HR) were assessed in both MT and active comparison groups. Only the MT group demonstrated significant improvements in HRV and reductions in HR, highlighting physiological benefits linked to MT.

As research continues to advance, maintaining high standards in study design will be critical for accurately assessing MT’s potential and ensuring its applicability across military and high-stress professional settings. In addition, future research should prioritize comprehensive assessments that capture MT’s impact across multiple metrics. This need is underscored by prior studies that have found mixed results, where MT demonstrated benefits in some areas but not others. For example, Yan et al. [80] found that while MT led to improvements in resilience, it did not yield significant changes in PTSD scores. Similarly, in one study [21], MT enhanced mental toughness and marksmanship under physical stress, but validated screening tools for depression and anxiety showed no significant group-by-time effects. In contrast, another study [16] reported MT-related improvements in depression and sleep but not in anxiety. This pattern of mixed and inconsistent findings across studies highlights the complexity of evaluating MT outcomes and the need for further research to clarify when and for whom MT is most effective. Variability in results may stem from factors such as the specific MT intervention employed [46], the measures used, the characteristics of the population studied, the timing of assessments [46], the delivery format of the training [65], and the degree to which the trainer is familiar with the military context [50].

## Implementing Mindfulness Training in the Military Context

While more research is needed, the growing number of positive findings have prompted calls for MT to be incorporated into traditional military training to support service members in meeting the demands of future operations [see 81, 82]. Yet there are practical considerations that must be addressed when implementing MT as a universal intervention. Moreover, implementation needs to take into account that individuals in a workplace setting like the military may be directed to participate in MT, in contrast to studies with civilians in which samples typically self-select.

**Fig. 3:**
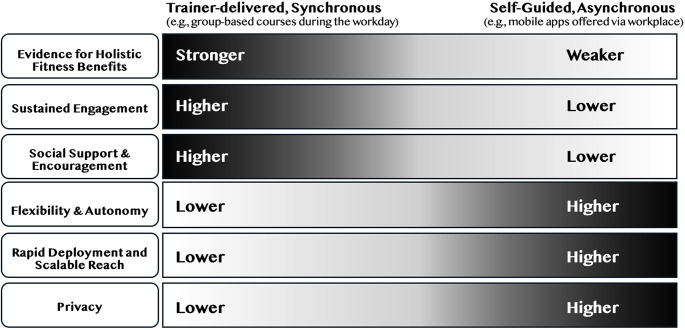
Considerations for Mindfulness Training Delivery in the Workplace

In civilian workplace settings, MT and other workplace wellness promotion programs have seen growing consensus regarding the strengths and weaknesses of specific implementation approaches [83]. For example, there are acknowledged trade-offs between live, trainer-facilitated program delivery versus asynchronous digitally supported delivery, via mindfulness apps (See Fig. 3). Whereas trainer-facilitated MT delivery has a stronger evidence-base and greater participant engagement, apps provide greater scheduling flexibility and privacy. In addition, military implementation of MT must also consider military culture, scheduling constraints, and whether MT enhances service members’ holistic fitness [see 14].

Many MT studies in the civilian and clinical context have used gold standard MT programs like Mindfulness Based Stress Reduction [MBSR, see 84]. While these MT programs have numerous strengths, the key features of these interventions may challenge their feasible implementation in an occupational context like the military. First, these MT programs involve a considerable time commitment. MBSR typically takes 24 h over an 8-week period and requires an additional 45 min of MT exercise outside of class per day that is expected to be maintained after the course ends. Second, the material is typically contextualized for stress and symptom reduction rather than performance improvement and may be seen as less relevant by service members. Third, there is a substantial and intense train-up for MBSR trainers that can take a year or longer. In addition, typical MT programs may not include specific guidance for how mindfulness practice can be integrated into moments during day-to-day life. These features of implementation (time, contextualization, trainer preparation, and practice) need to be addressed directly to create an approach for implementation and sustainable uptake in the military [see 14].

In order to create a program that is scalable within the military, it is important to conduct effectiveness studies to determine best practices. One way to accelerate the development of such empirical insights is to hold evaluation metrics constant and vary specific program parameters. Given the critical role of attentional control, its vulnerability to degradation under high demand circumstances, and its trainability with MT, attentional control variables may be a useful starting point (see Fig. 2). By focusing on attentional control variables, research on MT program effectiveness can examine questions about what features of MT programs are necessary to bring about salutary effects rather than on questions about whether a particular outcome is impacted by MT.

To that end, a series of MT feasibility trials have examined attentional control variables (e.g., sustained attention and working memory tasks) [see 19, 50]. Consistent with MT studies in civilians [27], initial military studies offering a longer format MT program [e.g., 20–24 hours; 23, 46] found benefits for attention and working memory performance. Importantly, in terms of developing empirically-driven implementation guidance, these benefits remained when the program time was shortened to 16 hours [19]. To determine if the program could be further shortened, two 8-hour, 8-week variants of the same program were offered during a high-demand pre-deployment interval [68, 69]. These two variants differed in training emphasis: one was focused on practice, with the bulk of class time spent engaging in mindfulness and practice-related discussion, the other was focused on didactics, emphasizing conceptual content regarding stress and symptom reduction. The practice-focused group demonstrated greater protection from performance decline in sustained attention [68] and working memory [69] compared to the didactic-focused group and the no-training control group. This 8-hr program yielded similar effects to the 16-hr MT program variant [70].

These results were encouraging regarding the feasibility of offering short-form MT to military populations to protect against cognitive decline over high-demand intervals. Yet, it is important to note that across these studies, the MT program was delivered by a single trainer who had also developed the MT program [76]. This trainer had extensive prior expertise in mindfulness practice, as well as familiarity with military service [see 85]. Such background aligns with recommendations that trainers have expertise in mindfulness-related training as well as knowledge, experience, and professional training related to the specific populations to whom MT will be delivered [86]. Realistically, however, individuals with such combined expertise and experience are rare, and the paucity of eligible trainers poses a challenge for rapid dissemination of MT to military groups.

In response to this challenge, one research team has developed a fast-tracked, 30-hour train-the-trainer (TTT) practicum which is delivered over a 10-week period. At the end of the practicum, trainers are evaluated for their readiness to deliver the 4-week, 8-hour MT program, and participants who subsequently receive the program from these trainers are also assessed. Using this approach, Army sport psychology experts have effectively trained soldiers [e.g., 16], military spouses have effectively trained peers [87], medical faculty have effectively trained medical students [88], and human resources professionals have effectively trained company employees [89]. In each case, studies using these trainers found positive effects associated with MT [16–18, 21, 50, 87, 89, 90].

These MT studies have also examined the pivotal role of out-of-class mindfulness practice to determine whether the amount and frequency of practice are linked to outcomes. Specifically, researchers have investigated whether a dose-response relationship exists and whether a minimum effective dose can be identified. Indeed, findings suggest that spending more time engaging in foundational practices such as focused attention, body scan, open monitoring, and connection is associated with stronger benefits [65]. In addition, practicing mindfulness exercises, provided through 15-minute audio recordings, at least 3 to 4 times per week over a four-week interval has been suggested as a threshold for observing measurable improvements [21]. In addition, beneficial outcomes have been reported when individuals incorporate brief mindfulness moments throughout the duty day, indicating that both formal and informal practice may contribute to the overall effectiveness of mindfulness training [16].

As a whole, MT research has demonstrated that training that follows best-practice implementation guidelines is effective. Specifically, MT programs offered to military cohorts that have been delivered in a time-efficient manner, provided by trainers familiar with the context, and focused on embedding mindfulness practices that can be integrated into daily life [16] are linked with better outcomes across all the relevant domains. Although certain program elements have been associated with better outcomes, there is variability in what MT programs are being disseminated and evaluated across various militaries. MT programs in active-duty populations include variants of MBSR such as MBSR-military cadets [15]; Team Mindfulness Training [64]; Mind–Body Medicine [91]; mindfulness-based mind fitness training [23], Stärken- und Ressourcentraining [Strength and resource training] [20], and mindfulness-based attention training [50]. Such a diverse list is a testament to how MT is being adapted for a variety of contexts within the military.

Still, military culture itself needs to be considered when implementing MT [14]. Military culture tends to prioritize being physically and mentally tough. At first glance, these cultural dimensions might appear inconsistent with MT. Those new to MT might assume MT is associated with being passive or weak. Yet those who are familiar with warrior traditions of stoicism will recognize the link between MT and mental discipline [92]. Indeed, MT is well-suited to military culture because it may strengthen a service member’s ability to focus attention on the mission [49]. In addition, military culture can facilitate MT uptake if MT takes a strength-based approach, highlights its relevance to military readiness, incorporates teams and leaders, and integrates MT practices into military exercises [93].

## Conclusions

The evidence for MT demonstrates its relevance to settings like the military, where individuals need to perform optimally under high-stress conditions that are likely to deplete their attentional resources. MT is easily portable and can be adapted to a variety of military contexts. Indeed, research has documented the benefits of MT, particularly when service members engage in routine practice. Furthermore, there have been advancements in ensuring effective MT dissemination using methods that are more readily scalable than traditional approaches.

Despite these advances, there are still ways in which scalability and sustainability can be improved. Future research can examine how the training pipeline can be further streamlined using alternative methods of MT delivery such as web courses and apps, while deliberately countering the tendency for app utilization to decline with time (see Fig. 3). Future research can also examine how guidance on enacting mindfulness during daily life might be more seamlessly integrated, given the demonstrated potential of this approach [16]. Rather than be considered a discrete military program, MT may be best considered as part of military life much like its counterpart of PT.

There are also many ways to introduce MT into organizational culture. Arguably, the most powerful is [94] by having leaders set the conditions, lead by example, educate and encourage, as well as plan and prioritize MT engagement [95, 96]. Previous studies have demonstrated the positive impact of this approach on the uptake of other health-related practices, including sleep [95] and family support [97] as well as occupational health promotion [98]. Indeed, leader behaviors that target health-related outcomes are associated with better employee outcomes above and beyond the contributions of general leadership skills [99]. In the case of MT, evidence suggests that new service members may benefit from being introduced to MT practices by leaders [94], while senior leaders may benefit by having MT crafted to emphasize its role in strategic thinking [100].

Besides tailoring MT to the developmental stage of the service member, any implementation plan would also have to tackle potential misunderstandings about the nature of MT. First, it would be critical to clarify that MT requires a minimum amount of practice to be effective. Thus, sprinkling exposure to MT practices in an ad hoc manner, or limiting the introduction of MT from an exclusively conceptual vantage point, without deliberate practice, are likely to be ineffective. These approaches can also potentially backfire as individuals who experience this kind of introduction to MT may lose their motivation and perceive MT as “just another mandatory training”.

Second, implementation would need to consider whether there are cultural biases against MT. There may be, for example, underlying beliefs that MT is not an appropriate fit for occupations that require performing in the face of extreme stress. These beliefs would need to be directly addressed through examples and research documenting the role of MT in promoting performance [21] and tolerance of distress [7, 101].

Future research can also address limitations in previous studies such as small sample size, MT content being underspecified, and engagement in exercises not being tracked. Furthermore, the metrics are variable. While that can demonstrate the breadth of impact, it is difficult to build a body of evidence regarding any one particular outcome for meta-analyses. Finally, effect sizes are typically small [13]. Nevertheless, it is common for applied research to find small effect sizes because there is greater error in the model [102]. In addition, similar studies of positive psychology interventions do not typically find large effect sizes. For example, a 2021 meta-analysis of 347 universal positive psychology interventions found small to medium effects [103]. From a public health perspective, even small effect sizes may have practical significance when the intervention is universally applied and effects may accumulate over time.

Besides examining questions of implementation, it would be helpful for future research to assess long-term effects of MT on the operational performance of individuals, teams and leaders using a holistic fitness approach to outcomes. It would also be useful to compare MT alone to MT integrated into other training programs in order to boost the effects of MT. Such results would provide a clearer path for future efforts.

Military MT research is in the early stages of development [13] and should be understood as such. Rather than criticize prior research as being poor quality, it can be helpful to consider prior research as establishing the foundation from which the next steps in research can emerge. While larger randomized controlled trials should occur, it can also be helpful to lean on the civilian MT literature that emphasizes efficacy and instead focus on pragmatic questions of implementation. Pragmatic trials are also useful when the context, like the military, requires research efforts to adapt to the constraints of a real-world occupational setting.

In the future, MT may be as ubiquitous as PT. Individuals may be introduced to MT when they enter basic combat training and continue practicing mindfulness with their first unit of assignment. They may broaden how they utilize MT as they develop professionally and as the demands on their attention change. Ultimately, the scene described at the start of this article—service members finishing an early morning run and seamlessly transitioning into a 15-minute mindfulness exercise—may become standard practice. And if it does, it may not only bolster individual service members’ holistic fitness but, by enhancing their cognitive sharpness, social connection, and emotional balance, provide militaries that offer a daily opportunity to practice mindfulness exercises with a decisive edge—especially in a future expected to be increasingly complex, uncertain, and operationally demanding.

## Key References

- Draheim, C., R. Pak, A.A. Draheim, & R.W. Engle. (2022). The role of attention control in complex real-world tasks. *Psychon Bull Rev*, *29*(4), 1143–1197. 10.3758/s13423-021-02052-2. **This review examined research on attentional control and working memory, highlighting their importance for educational achievement, athletic performance, and psychological health.**
- Lieberman, H.R., G.P. Bathalon, C.M. Falco, F.M. Kramer, C.A. Morgan, 3rd, & P. Niro. (2005). Severe decrements in cognition function and mood induced by sleep loss, heat, dehydration, and undernutrition during simulated combat. *Biol Psychiatry*, *57*(4), 422–429. **This seminal study assessed the impact of an intensive interval of military field training on elite service members. Over the interval**,** they experienced significant cognitive and mood impairments attributed to the intensive demands of their training.**
- Zainal, N.H., & M.G. Newman. (2024). Mindfulness enhances cognitive functioning: a meta-analysis of 111 randomized controlled trials. Health Psychol Rev, 18(2), 369–395. 10.1080/17437199.2023.2248222. **This meta-analysis of 111 RCTs found that mindfulness-based interventions produced improvements in global cognition and specific cognitive subdomains**,** such as attentional control**,** with stronger effects observed in studies with face-to-face delivery.**
- Sezer, I., D.A. Pizzagalli, & M.D. Sacchet. (2022). Resting-state fMRI functional connectivity and mindfulness in clinical and non-clinical contexts: A review and synthesis. *Neurosci Biobehav Rev*, *135*, 104,583. 10.1016/j.neubiorev.2022.104583. **This review highlighted evidence that MT leads to altered connections in key brain networks involved in attention**,** self-awareness**,** emotion regulation**,** and pain relief.**
- Hepner, K.A., E.L. Bloom, S.J. Newberry, J.L. Sousa, K.C. Osilla, M. Booth, A. Bialas, & C.M. Rutter. (2022). *The Impact of Mindfulness Meditation Programs on Performance-Related Outcomes: Implications for the U.S. Army*. RAND Corporation. 10.7249/RRA1522-1. ** This systematic review examining the implications of mindfulness meditation research for the US Army found that mindfulness meditation may improve attention**,** emotion regulation**,** impulse control**,** and reduce stress and recommended further research to identify best practices for implementing mindfulness programs with US Army soldiers.**
- Nassif, T.H., A.L. Adrian, I.A. Gutierrez, A.C. Dixon, S.L. Rogers, A.P. Jha, & A.B. Adler. (2023). Optimizing Performance and Mental Skills With Mindfulness-Based Attention Training: Two Field Studies With Operational Units. *Military Medicine*, *188*(3–4), e761-e770. 10.1093/milmed/usab380. **This study evaluated the impact of a short-form mindfulness training program on operational performance and mental skills in active-duty soldiers through two randomized trials. Results showed that practicing three or more days per week was associated with improved marksmanship under stress**,** fewer attentional lapses**,** better emotion regulation**,** greater mental toughness**,** and higher self-reported mindfulness.**
- Nassif, T.H., I.A. Gutierrez, C.D. Smith, A.P. Jha, & A.B. Adler. (2023). The Effect of a Combined Mindfulness and Yoga Intervention on Soldier Mental Health in Basic Combat Training: A Cluster Randomized Controlled Trial. *Depression and Anxiety*, *2023*, 1–11. 10.1155/2023/6869543. **This study examined the effects of a mindfulness and yoga intervention on the mental health of soldiers-in-training during Basic Combat Training**,** finding that the intervention led to a greater reduction in depression and sleep problems compared to training-as-usual. While the study could not isolate the effects of each component**,** the results suggest that mindfulness and yoga may help maintain mental health during high-stress training.**
- Jha, A.P., A.P. Zanesco, E. Denkova, W.K. MacNulty, & S.L. Rogers. (2022). The Effects of Mindfulness Training on Working Memory Performance in High-Demand Cohorts: a Multi-study Investigation. J Cogn Enhanc, 6(2), 192–204. 10.1007/s41465-021-00228-1. **This internal meta-analysis found that MT helps protect working memory in military personnel under stress. Across six studies**,** those who received MT showed moderate improvements in working memory compared to control groups**,** suggesting MT can enhance cognitive resilience in demanding environments.**
- Jha, A.P., A.P. Zanesco, E. Denkova, A.B. Morrison, N. Ramos, K. Chichester, J.W. Gaddy, & S.L. Rogers. (2020). Bolstering Cognitive Resilience via Train-the-Trainer Delivery of Mindfulness Training in Applied High-Demand Settings. *Mindfulness*, *11*(3), 683–697. 10.1007/s12671-019-01284-7. **This study tested a “train-the-trainer” model for delivering short-form MT to military service members. Three groups were compared: one trained by military-familiar trainers**,** one by a mindfulness expert unfamiliar with the military**,** and a no-training control. All groups showed declines in attention and working memory over a military readiness training interval**,** but the military-familiar trainers’ group experienced smaller declines**,** highlighting the benefits of context familiarity in MT delivery.**
- Zanesco, A.P., E. Denkova, S.L. Rogers, W.K. MacNulty, & A.P. Jha. (2019). Mindfulness training as cognitive training in high-demand cohorts: An initial study in elite military servicemembers. Prog Brain Res, 244, 323–354. 10.1016/bs.pbr.2018.10.001. **This randomized controlled study of mindfulness training in elite military service members found greater benefits to sustained attention and working memory performance following delivery over a 4-week interval relative to a 2-week interval.**

## Data Availability

No datasets were generated or analysed during the current study.

## Declarations

### Author Note for AA

Material has been reviewed by the Walter Reed Army Institute of Research. There is no objection to its presentation and/or publication. The opinions or assertions contained herein are the private views of the author, and are not to be construed as official, or as reflecting true views of the Department of the Army or the Department of Defense.
